# Exploration of correlation of oral hygiene and condition with influenza infection

**DOI:** 10.1371/journal.pone.0254981

**Published:** 2021-08-18

**Authors:** Makiko Kawamoto, Hirokazu Tanaka, Akinari Sakurai, Hiroki Otagiri, Imahito Karasawa, Shin-ichi Yamada, Hiroshi Kurita

**Affiliations:** Department of Dentistry and Oral Surgery, Shinshu University School of Medicine, Matsumoto, Japan; Montana State University, UNITED STATES

## Abstract

Influenza viruses are known to be infected through epithelial cells of the upper respiratory tract. The oral cavity is in close anatomical proximity to the upper respiratory tract, and it is conceivable that the viruses could pass through the oral cavity and infect to the upper respiratory tract. Several researchers have suggested that colonization of certain pathogenic bacteria such as Staphylococcus aureus or Streptococcus pneumoniae might affect the risk of influenza viral disease, indicating that oral hygiene and/or condition might play an important role in respiratory viral infection. Therefore, the purpose of this study was to investigate whether an oral hygiene/condition might impact influenza infection. We conducted a retrospective observational study of Japanese citizens’ regional cohort (N = 2,904) consisting of National Health Insurance beneficiaries who underwent annual health/dental examination with data entries in the Kokuho database (KDB). Trained dentists checked the oral hygiene/condition, and saliva specimens were examined using the LION dental saliva multi-test (SMT) kit. Influenza infection was identified from the diagnosis recorded in the KDB. The correlations between influenza infection and oral hygiene, dryness of the mouth, or various salivary test results were examined by a multivariate analysis adjusting for confounding factors such as gender, age, recent smoking, alcohol drinking, BMI, HbA1c, RBC for influenza infection. The logistic regression model showed that age significantly correlated with influenza infection. In addition, oral hygiene status had a nearly significant impact on influenza infection (p = 0.061), whereby, the subjects with poor oral hygiene had a higher risk of influenza infection than those with good oral hygiene (odds ratio: 1.63, 95% confidence interval: 0.89–2.95). Further, the prevalence of influenza infection was lower in the subjects with saliva weakly acidic and/or containing higher protein level. The results of this study suggested that the maintenance of oral health conditions might be one of the pivotal factors for preventing and reducing influenza infection.

## Introduction

Influenza (seasonal and pandemic) is an infectious disease of the respiratory tract caused by influenza (flu) viruses, with a high mortality rate in the elderly. Most people exposed to the influenza virus are asymptomatic, without noticeable symptoms, while a small minority develop symptoms, signs, and/or experience severe illness. Variation in susceptibility could be extremely broad. A variety of factors are reported as host susceptibility for influenza infection, including previous exposure of viruses, vaccination, and individual’s demographic and clinical characteristics (age, gender, obesity, nutrition or medicine immunosuppression, chronic comorbidity such as chronic immunosuppression, cardiovascular disease, and neuromuscular diseases, along with pregnancy, microbiome, genetics, and so on) [1–3]. Prediction and control of these risk factors might contribute to the risk reduction of influenza infection.

Usually, influenza viruses primarily infect epithelial cells of the upper respiratory tract [4]. The reason for this is that influenza viruses strongly prefers to use α2,6-linked sialic acids present on the surface of the upper respiratory as receptors. The presence of sialic acid residues in the oral cavity has been reported [5], but there are no reports of a α2,6-linked sialic acids in the oral cavity. Previously Abe et al. reported that maintenance of oral hygiene is effective in the prevention of influenza infection in the elderly [6]. These results suggested that deterioration of oral hygiene/condition might lead to increased risk for susceptibility to influenza infection. Therefore, the purpose of this study was to investigate if oral hygiene/condition could impact influenza infection. To address this issue, we observed the prevalence of influenza infection among Japanese citizens who underwent assessment of oral hygiene/condition by retrieving data from the National Health Insurance database, which included participants demographics, laboratory tests, oral health assessment data, which were subjected to analyses and variables comparisons.

## Participants and methods

### Participants enrollment

We carried out a retrospective observational cohort study using data from health/dental examinations and the Japanese National Health Insurance database [7] (Kokuho database; KDB) of the subjects who underwent annual group-specific dental health checkups in Shiojiri city, Japan from September 2017–April 2019. Our inclusion criteria were community-dwelling residents. “Kokuho” covered the medical insurance of self-employed workers, farmers, retirees, and their dependents of this community. The KDB keeps records of almost all information regarding patients’ diagnoses, along with inpatient and outpatient medical and dental service use. The exclusion criteria were data from pregnant women because pregnancy is thought to be a risk factor for influenza infection [8] and individuals who were eating and/or drinking, teeth brushing, or had gargled 2 hours before the salivary examination.

### Ethical considerations

The protocol of the present study was approved by the Committee on Medical Research of Shinshu University, Japan (Approval ♯4815). The subjects all provided written informed consent to collect and use demographics and clinical data from the health/dental examination and the KDB, and the data from the informed and consented individuals were used for the analysis. The study was conducted following the ethical principles of the Helsinki Declaration update of 2008.

### KDB data collection

Patients’ demographics data and medical records were retrieved from KDB (the last access on 2019/8/30). Influenza infection was identified from the diagnosis recorded in the KDB. In Japan, the diagnosis of influenza is usually made with an influenza antigen qualitative test kit (influenza A or B). Specific health checkup had been conducted following standard program provided by the Ministry of Health, Labor and Welfare of Japan [9]. It included an interview on lifestyle (including recent smoking habit and alcohol drinking), measurement of height, body weight, abdominal circumference, blood pressure, and blood tests (triglyceride, low/high-density lipoprotein cholesterol, hemoglobin A1c (HbA1c) levels, red blood cell (RBC) counts, and creatinine). Dental checkup included an inspection of dental and periodontal tissues, as well as oral hygiene and dryness of the mouth, assessed by trained dentists. Oral hygiene status was classified into three grades (good: clean and no food particles or tartar in mouth or dentures, fair: food particles/tartar/plaque in 1–2 areas of the mouth or small area of denture or halitosis, and poor: food particles/tartar/plaque in most areas of the mouth or on most of the dentures or severe halitosis), according to the buccal cavity cleanliness of Oral Health Assessment Tool [10]. The dryness of the mouth was classified into four grades according to the clinical classification reported by Kakinoki (normal: non-dry, slight: saliva shows viscosity, moderate: saliva shows tiny bubbles on the tongue, and severe: dry tongue with little or no saliva present) [11].

### Salivary examination

In 2017 and 2018, a salivary examination was carried out in addition to the dental checkup. Prior to the dental examination, a salivary sample was collected with 3 ml of mouthwash liquid and immediately evaluated using a commercially available test kit, Salivary Multi-Test (SMT) (LION Dental Products Co., Ltd., Tokyo, Japan) [12]. SMTs were performed according to the manufacturer’s protocol. SMT evaluated each level of cariogenic bacteria, acidity, buffer capacity, occult blood, leukocyte count, protein, and ammonia in saliva. This system consists of test strips and a measuring device. It detects color changes in the test strip and measures the reflectance by specified wavelength. The cariogenic bacteria reflect the reduction ability of resazurin sodium by gram-positive bacteria, the acidity demonstrates a change in the coloration of a pH indicator; the acid buffering capacity reflects the coloration change of the compound pH indicator under the fixed quantity of acid existence. Occult blood was detected by determining hemoglobin pseudo-peroxidase activity as an index. Leukocyte level was detected by the measurement of esterase activity in isolated salivary leukocyte. The protein-error error of indicators reaction was used to determine protein levels. Ammonia was detected by a color change of bromocresol green [13–15]. The principle of the detection of the SMT and detection range are summarized in S1 Table; the values of the test results were expressed as a relative value (percentage; 0–100% within the detection range), and the final 3 ranks of classification (high, average, or low) was achieved according to reference value established by the manufacturer [16].

### Statistical analyses

The correlations between influenza infection and either oral hygiene, dryness of the mouth, or the results of the salivary examination were assessed using uni- and multivariate analysis. In multivariate analysis, the logistic regression model was employed, including gender, age, recent smoking, alcohol drinking, BMI, HbA1c, RBC count as possible cofounding risk factors for influenza infection. Chi-square test was used to assess the differences between categorical variables within test groups. The student t-test was employed to determine the differences between the means of the variables measured. Mann-Whitney U-test was utilized to test whether the values between groups were statistically significant. A p value of less than 0.05 (p < 0.05) was considered significant. All statistical analyses were performed using JMP ver.13 (SAS Institute Inc., North Carolina, USA).

## Results

The primary outcome measure of the present study was an influenza infection. Influenza infection was identified from the diagnosis recorded in the KDB. Each subject was followed-up for influenza infection through the same fiscal year (from April to March) as the health/dental checkup was carried out (September to January). In Japan, influenza activity most commonly peaks between December and March. Among individuals who underwent the specific health checkup, 906 (36.5%) out of 2,485 in 2017, 1,097 (43.5%) out of 2,519 in 2018, and 901 (37.1%) out of 2,426 provided consent, making a total of N = 2,904 enrollment of participants for the study. There was a total of 1,513 (52.1%) female and 1,391 (47.8%) male with age ranging from 30–94 years (Table 1). Clinical diagnosis of influenza was found in 43 subjects in 2017, 59 in 2018, and 57 in 2019, with a prevalence rate of 4.7%, 5.4%, and 6.3%, respectively.

**Table 1:** Characteristics of the studied subjects.

		2017		2018		2019		Total	
		n	%	n	%	n	%	n	%
**Number**		906	ー	1,097		901	ー	**2,904**	**ー**
**Gender**	**Female**	494	54.5%	569	51.9%	451	50.1%	**1,513**	**52.1%**
	**Male**	412	45.5%	529	48.2%	450	49.9%	**1,391**	**47.9%**
**Age**	**30–39**	78	8.6%	78	7.1%	99	11.0%	**255**	**8.8%**
	**40–49**	109	12.0%	131	11.9%	84	9.3%	**324**	**11.2%**
	**50–59**	115	12.7%	136	12.4%	114	12.7%	**365**	**12.6%**
	**60–69**	395	43.6%	424	38.7%	330	36.6%	**1,149**	**39.6%**
	**70–79**	200	22.1%	308	28.1%	253	28.1%	**761**	**26.2%**
	**80-**	9	1.0%	20	1.8%	21	2.3%	**50**	**1.7%**
**Subject who underwent salivary examination**		886	97.8%	1,088	99.2%	ー	ー	**1,974**	**68.0%**
**Influenza infection**		43	4.7%	59	5.4%	57	6.3%	**159**	**5.5%**

The result of the univariate analyses, which assessed the correlation between influenza infection and possible risk factors, including dryness of the mouth and oral hygiene status, are shown in Table 2. There was a statistically significant correlation between influenza infection and age. The mean age was lower in the subjects with influenza infection than those without (Student t-test, p < 0.01). Besides, there was a nearly significant relationship between influenza infection and dryness of the mouth (p = 0.072).

**Table 2:** Result of univariate analyses assessing the correlation between influenza infection and risk factors including oral condition.

		Diagnosis with influenza		Results of univariate analysis	
		Yes (159)	No (2,745)	p-value	Test name
**Year**	**2017**	43 (4.8%)	863	0.334	Chi-square test
	**2018**	59 (5.4%)	1,038		
	**2019**	57 (6.3%)	844		
**Gender**	**Female**	82 (5.4%)	1,431	0.891	Chi-square test
	**Male**	77 (5.5%)	1,314		
**Age (years)**	*Ave*. *+- SE*	56.09 +- 0.99	61.59 +- 0.24	< 0.01	Student t-test
**BMI (kg/m2)**	*Ave*. *+- SE*	23.23 +- 0.28	22.85 +- 0.07	0.191	Student t-test
**HbA1C (%)**	*Ave*. *+- SE*	5.66 +- 0.05	5.72 +- 0.01	0.202	Student t-test
**RBC (x 10000 /μL)**	*Ave*. *+- SE*	472.05 +- 3.31	467.55 +- 0.80	0.189	Student t-test
**Recent smoking**	**Yes**	20 (6.6%)	281	0.349	Chi-square test
	**No**	139 (5.4%)	2,459		
**Alcohol drinking**	**Every day**	42 (6.4%)	614	0.314	Mann-Whitney u-test
	**Chance**	44 (5.3%)	780		
	**No**	73 (5.2%)	1,340		
**Dryness of the mouth**	**Normal**	152 (5.7%)	2,526	0.072	Mann-Whitney u-test
	**Slight**	5 (2.7%)	180		
	**Moderate**	1 (3.5%)	28		
	**Severe**	0 (0.0%)	2		
**Oral hygiene status**	**Good**	54 (4.8%)	1,068	0.203	Mann-Whitney u-test
	**Fair**	88 (5.8%)	1,423		
	**Poor**	17 (6.3%)	254		

Additionally, the result of the multivariate analysis assessing the correlation between influenza infection and the oral condition is summarized in Table 3. The logistic regression model showed that younger age was a significant independent risk for influenza infection. In addition, the oral hygiene status had a nearly significant impact on influenza infection (p = 0.061). The subjects with poor oral hygiene had a higher risk of influenza infection than those with good oral hygiene (odds ratio: 1.63, 95% confidence interval: 0.89–2.95).

**Table 3:** Result of multivariate analysis assessing the correlation between influenza infection and oral condition.

	Estimate	Standard error	t-value	p-value
**Year [2017/2019]**	-0.173	0.124	1.94	0.163
**Year [2018/2019]**	0.030	0.115	0.07	0.798
**Gender (Male/Female)**	-0.084	0.104	0.65	0.420
**Age (years)**	-0.032	0.007	23.5	< .0001
**BMI (kg/m2)**	0.026	0.023	1.28	0.259
**HbA1c (%)**	0.005	0.152	0.00	0.975
**RBC (count/μL)**	0.000	0.002	0.03	0.873
**Recent smoking (Yes/No)**	-0.056	0.134	0.18	0.674
**Alcohol drinking** ^*1^	-0.137	0.111	1.53	0.216
**Dryness of the mouth** ^*2^	-0.520	0.368	1.99	0.158
**Oral hyginene status** ^*3^	0.256	0.137	3.51	0.061

*1: No/Chance/Every day.

*2: Severe/Moderate/Slight/Normal.

*3: Bad/Fair/Good.

Further, the correlation between influenza infection and the results of the salivary examination is summarized in Table 4. Multivariate analyses showed the significant relationship between influenza infection and either saliva acidity, revealing prevalence (High: 4.5%, Avg: 5.8%, and low: 8.2%, Estimate -0.397,p < 0.01) or protein level (High: 3.7%, Avg: 6.4%, and low: 7.2%, Estimate -0.285, p <0.05), revealing a lower prevalence of influenza infection in the subjects with high saliva acidity (low pH) and/or high protein level. While, no statistically significant relationship was observed regarding cariogenic oral bacteria, buffer capacity, occult blood, leucocytes, and ammonia with influenza infection (Table 4).

**Table 4:** Result of uni- and multivariate analysis assessing the correlation between influenza infection and results of salivary examination.

		influenza infection		Results of univariate analysis		Results of multivariate analysis*			
		Yes (102)	No (1,879)	p-value	*Test name*	Estimate	SE	t-value	p-value
**Cariogenic bacteria**	**High**	39 (4.8%)	776	0.143	*Mann-Whitney u-test*	-0.145	0.123	1.39	0.238
	**Average**	20 (3.8%)	502						
	**Low**	43 (6.7%)	601						
**Acidity**	**High**	60 (4.5%)	1,277	< 0.05	*Mann-Whitney u-test*	-0.397	0.144	7.55	< 0.01
	**Average**	26 (5.8%)	422						
	**Low**	16 (8.2%)	180						
**Buffer capacity**	**High**	30 (4.6%)	617	0.248	*Mann-Whitney u-test*	-0.057	0.138	0.17	0.682
	**Average**	34 (4.8%)	675						
	**Low**	38 (6.1%)	587						
**Occult Blood**	**High**	37 (4.2%)	850	0.126	*Mann-Whitney u-test*	-0.141	0.132	1.15	0.284
	**Average**	40 (6.1%)	615						
	**Low**	25 (5.7%)	414						
**Protein**	**High**	38 (3.7%)	985	< 0.01	*Mann-Whitney u-test*	-0.285	0.135	4.46	< 0.05
	**Average**	37 (6.4%)	546						
	**Low**	27 (7.2%)	348						
**Leukocyte**	**High**	42 (4.4%)	918	0.052	*Mann-Whitney u-test*	-0.220	0.130	2.85	0.091
	**Average**	32 (5.1%)	602						
	**Low**	28 (7.3%)	359						
**Ammonia**	**High**	79 (5.2%)	1,452	0.959	*Mann-Whitney u-test*	0.177	0.191	0.86	0.353
	**Average**	17 (5.2%)	312						
	**Low**	6 (5.0%)	115						

*Cofounding factors included in the multivariate analysis: Year (2017, 2018), Gender, Age (years), BMI (kg/m2), HbA1c (%), RBC (x 10000 /μL), Recent smoking (Yes/No), Alcohol drinking (Every day/Chance/No).

## Discussion

In this study, the incidence of influenza infection and its correlation with oral hygiene was analyzed using a cohort from one of the Japanese National Health Insurance databases, KDB, which covers all influenza diagnosis and treatment for the beneficiaries, as well as the results of medical and dental checkup. The results suggested that oral hygiene status might be one of the risk factors that explain influenza infection. Multivariate analysis revealed a nearly significant correlation between oral hygiene status and influenza infection. The influenza infection was more prevalent in subjects with poor oral hygiene than those with good oral hygiene. The relative risk for acquiring influenza infection was calculated as 1.31 (poor: 6.2%/good: 4.8%). Previously Abe et al. studied the effect of professional oral care in the elderly and related outcomes to the prevention of influenza infection. Their results showed that the maintenance of oral hygiene was effective in the prevention of influenza [6]. Our result was consistent with their finding.

Influenza viruses present two major surface glycoproteins, the haemagglutinin (HA) and the neuraminidase (NA), that play a crucial role in influenza entry, exit and replicative infection [17–19]. These two glycoproteins recognize the same host cell molecule: the sialic acid (SA) (generic term for the N- or O-substituted derivatives of neuraminic acid) and have a complementary role in the replication cycle. The HA initiates the virus entry by binding to the SA and the NA facilitates the virion release from infected cells through its sialidase activity [20]. Membrane fusion and subsequent infectivity are dependent upon cleavage of the HA precursor molecule. Proteolytic cleavage of HA is required for cell entry by receptor-mediated endocytosis and plays a key role in pathogenicity of the virus. It has been reported that coexisting bacteria could play an essential role in the development of influenza infection by providing a protease suitable for cleavage activation of the HA [21]. Some bacteria have been found to secrete proteases such as trypsin-like proteases (TLP) and others that activate the infectivity of the influenza virus via proteolytic cleavage of the HA [22]. Abe et al. showed that professional oral care reduced the oral bacterial count, as well as the TLP activity [6]. These results suggest that professional oral care might help reduce the risk of influenza infection by reducing the number of bacteria in the mouth. Besides, NA on the surface of the virus also plays an essential role in infection and release of the replicated virus [23]. Abe et al. also showed that the professional oral care had a significant effect in reducing NA activity [6]. From these results we speculated that poor oral hygiene might contribute to infection and increase of influenza virus concentration in the oral cavity, which might become a reservoir for severe infectious diseases.

Further, it has been reported that secondary bacterial infection in influenza is frequent in critically ill patients [24], and it is thought that this infection with influenza disrupts the respiratory tract via the direct pathogenic attack of the alveolar cells, which then predisposes the lung to secondary bacterial pneumonia. Recent evidence has shown a critical role of the oral bacteria in the process of respiratory infection [25–28]. It is evident that the oral cavity, along with the nasal cavity, being proximal to the airway, would be the most direct route for microbes to enter the airway [26]. Oral bacteria could be aspirated into the lung and cause pneumonia, especially in the elderly, dysphagia, and the medically compromised patients (pulmonary disease, degenerative neurologic diseases, ventilated patients, etc.) are most susceptible [25,28]. Some studies have suggested that the control of oral hygiene had a positive impact on reducing aspiration pneumonia [29–33]. Prevention and treatment of secondary bacterial infection should be an integral part of the influenza infection. Maintenance of good oral hygiene should be a crucial factor in preventing and treating severe influenza infection.

Moreover, studies have shown that the prevalence of influenza infection was lower in the subjects with saliva containing higher acidity (low pH). In this study, SMT was utilized to evaluate the properties of the saliva. The SMT evaluated the acidity of saliva within the range of pH 6–8 [15,16]. In the oral cavity, the pH was maintained near neutrality (6.7–7.3) by saliva, which has a normal pH range of 6.2–7.6, with 6.7 being the average.^30^ Therefore, our result suggested that influenza infection was less prevalent in the subjects with weakly acidic saliva than those with alkalescent salivation. The strict reason why weakly acidic saliva had a negative impact on influenza infection is unknown. However, it has been reported that the pH stability of HA affects the transmissibility of influenza viruses. Virus binding and uptake by a specific host cell are mostly determined by the composition of the receptor-binding site of the HA1 domain, whereas the fusion potential of HA correlated with its pH-dependent stability. The variation in pH sensitivity and acid stability of influenza virus has been reported [34–37]. Takahashi and Suzuki reported that for the human influenza A virus, NAs of all pandemic viruses were low-pH-stable, whereas those of almost all human seasonal viruses were not [38]. Seasonal influenza observed in this study might have less stability in weakly acidic environment. Hypochlorous acid (HOCl) has been shown to inactivate a variety of viruses, including coronaviruses and avian influenza viruses [39,40]. The evidence concerning the mechanism of HOCl was limited, but, in general, it denatures and aggregate proteins such as those of the viral capsid or surface compounds, and the lipid envelop [41]. HOCl also destroys nucleic acid via chlorination by forming chloramines and nitrogen-centered radicals [42]. Between pH levels of 3 and 6, the predominant species is HOCl (hypochlorite ion (OCl^−^) has the maximal antimicrobial properties [41,43]. These results suggested that salivary pH is one of the key factors against virus infection. This study also showed that the prevalence of influenza infection was lower in the subjects with saliva containing a higher amount of protein. Human saliva comprises 99.5% water, but also contains many vital substances, including electrolytes, mucus, antibacterial compounds, and various enzymes [43]. Protein in the saliva, such as mucins, lysozyme, and peroxidase, assume an essential role in lubrication, barrier function, and microbial interactions [44]. In addition, the antimicrobial peptides beta-defensins and cathelicidin LL-37 are also present in saliva in the oral cavity [45,46]. It is reasonable to think that a higher amount of protein in saliva would protect the oral epithelium from viral infection.

There are numerous risk factors involved in the determinant of influenza infection, such as individual characteristics (young age, female gender, low pre-epidemic antibody titer, vaccination, and others), environment (residence in an urban area, contact of infected individuals), and compliance to preventive measures (hand wash, social distancing) [1–3,47,48]. In this study, the effect of oral condition on influenza infection was analyzed considering the influence of other possible risk factors using multivariate analysis. However, the major limitation of this study is a lack of information concerning pre-existing antibody and vaccination against the influenza virus, which is thought to be major factors explaining infection. Unfortunately, the KDB had no data on past and current vaccination. And it had not been possible to interview subjects about their history of influenza infection and vaccination status because of retrospective nature of the study. There was a possibility that poor oral hygiene might link with seasonal influenza vaccine uptake. It is well known that influenza viruses constantly change through a process called antigenic drift [49], and there are several subtypes of influenza virus. Thus, the effectiveness of the host immune system and vaccine effectiveness differs according to the circulating type of seasonal influenza viruses in each year. Therefore, we observed the prevalence of influenza infection for three years and assessed the correlation between the oral condition and influenza infection. The result of this study showed that poor oral hygiene had a nearly significant impact on susceptibility for influenza infection regardless of the differences of the years with the different vaccine effectiveness (Seasons Vaccine Effectiveness Estimates in Japan [50], 41.3% (95%CI 25.2–54%) in 2017–18 and 30.4% (11.2–45.4%) in 2018–19). Abe et al. also reported that the maintenance of oral hygiene effectiveness in preventing influenza infection regardless of the vaccination of elderly subjects [6].

Our study does not preclude other limitations; it lacked information concerning participants’ social contacts and sociodemographic factors. Nonetheless, this study was carried out in Shiojiri city, a regional town with a population of about 67,000. Only self-employed workers, farmers, retires, and their dependents were invited and included in this study. Therefore, we considered that the influences of risk factors such as social contacts, socioeconomic status, and preventive measures were relatively low. In addition, it was unclear how many of the subjects had their natural teeth and how many had dentures. These may affect the type of bacteria present in the oral cavity, which might contribute to influenza virus susceptibility in this study. The effect of the different strains on influenza infection is unknown. We will study this in the future.

In conclusion, the results of this study suggested the possibility that influenza infection was more prevalent in the subjects with poor oral hygiene and that weakly acidic and lubricated (protein-rich saliva) oral conditions might promote the prevention of influenza infection. Maintenance of oral cleanliness and condition might be one of the pivotal factors in avoiding and reducing influenza infection. Very recently, the severe acute respiratory syndrome coronavirus 2 (SARS-CoV-2) has caused a pandemic of coronavirus disease of 2019 (COVID-19). Little is known about the interaction between SARS-CoV-2 and oral bacteria. It is reported that coronavirus needs ACE-2 expression in the oral cavity mucosa; thus, there is a potentially substantial COVID-19 infectious vulnerability risk for oral cavity [51], and microaspiration of oropharyngeal secretions are deemed responsible for severe respiratory diseases. Accordingly, maintenance of oral condition might be necessary for overcoming COVID-19.

## Supporting information

S1 Table Detection principle of the Salivary Multi Test. (XLSX)

S1 Data Minimum data 2904. (XLSX)
